# Selection for antimicrobial resistance is reduced when embedded in a natural microbial community

**DOI:** 10.1038/s41396-019-0483-z

**Published:** 2019-08-05

**Authors:** Uli Klümper, Mario Recker, Lihong Zhang, Xiaole Yin, Tong Zhang, Angus Buckling, William H. Gaze

**Affiliations:** 1 CLES & ESI, University of Exeter, Penryn, Cornwall, UK; 2 European Centre for Environment and Human Health, University of Exeter Medical School, ESI, Penryn, Cornwall, UK; 3 College of Engineering, Mathematics and Physical Sciences, University of Exeter, Penryn, Cornwall, UK; 4 Department of Civil Engineering, University of Hong Kong, Hong Kong, China

**Keywords:** Antibiotics, Microbial ecology, Bacterial evolution

## Abstract

Antibiotic resistance has emerged as one of the most pressing, global threats to public health. In single-species experiments selection for antibiotic resistance occurs at very low antibiotic concentrations. However, it is unclear how far these findings can be extrapolated to natural environments, where species are embedded within complex communities. We competed isogenic strains of *Escherichia coli*, differing exclusively in a single chromosomal resistance determinant, in the presence and absence of a pig faecal microbial community across a gradient of antibiotic concentration for two relevant antibiotics: gentamicin and kanamycin. We show that the minimal selective concentration was increased by more than one order of magnitude for both antibiotics when embedded in the community. We identified two general mechanisms were responsible for the increase in minimal selective concentration: an increase in the cost of resistance and a protective effect of the community for the susceptible phenotype. These findings have implications for our understanding of the evolution and selection of antibiotic resistance, and can inform future risk assessment efforts on antibiotic concentrations.

## Introduction

The emergence and spread of antimicrobial resistance (AMR) genes in bacterial pathogens has been identified as one of the major threats to human health by the World Health Organisation [1]. Whilst AMR genes have been detected in ancient permafrost samples [2], anthropogenic use of antibiotics has caused a rapid increase in their prevalence [3]. A large body of theory and in vitro work has identified the role of ecological context, such as treatment regime and environmental heterogeneity, in AMR gene dynamics [4–7]. However, the majority of this work has not explicitly considered a crucial feature of microbial ecology: microbes are typically embedded within complex communities of interacting species. This is always the case within human and livestock microbiomes, in which antibiotic-imposed selection is likely to be particularly strong [8]. Here, we combine experiments and theory to determine how selection for AMR is influenced by the presence of other species derived from a natural gut microbial community. The focus of this study is selection for pre-existing resistance genes within a focal species, rather than selection on de novo variation arising through spontaneous mutations or acquired through horizontal gene transfer from another species.

Recent experimental studies suggest that selection for AMR genes in complex communities is occurring at antibiotic concentrations (the minimum selective concentration; MSC) that are much lower than those that prevent the growth of susceptible bacteria (minimum inhibitory concentration; MIC) [9, 10]; as has been previously shown within single species in vitro [6, 7, 11]. However, it is unclear how the presence of other microbial species affects the MSC. While the precise effect of other species is likely context dependent, we hypothesise that the presence of the community will typically increase the MSC. Studies of single species suggest that resistant cells can afford protection to susceptible ones, through both, intracellular and extracellular degradation of antibiotics [12–14], thus increasing the relative fitness of susceptible strains and hence the MSC. However, excreted metabolites can both potentiate or decrease antibiotic efficacy, thus decreasing or increasing MSCs [15, 16]. Further, any costs associated with AMR may be enhanced by increased competition for resources, as, for example, has been observed with respect to resistance in flies to parasitoids [17] and bacteria to viruses [18].

To explore the potential effects of community context on AMR selection, we competed isogenic *Escherichia coli* MG1655 strains, differing exclusively in a single chromosomal resistance determinant, in the presence and absence of a microbial community across a gradient of two different aminoglycoside antibiotics, kanamycin (Kn) and gentamicin (Gm). We embedded the *Escherichia coli* (*E. coli*), commonly found in the anaerobic digestive tract of warm-blooded mammals [19], within a pig faecal community in experimental anaerobic digesters in an attempt to partially mimic a gut environment. We additionally employed metagenomic analysis, community typing (16S rRNA gene) and mathematical modelling to provide insights into mechanisms underpinning community effects on AMR selection.

## Material and methods

### Pig faecal community

Pig faeces were collected from four Cornish Black pigs without previous exposure to antibiotics in April 2016 on Healey’s Cornish Cyder farm (Penhallow, Cornwall, United Kingdom). Two hundred grams of faeces from each pig were pooled, mixed with 400 mL each of sterile glycerol and 1.8 g/L NaCl solution. The mixture was homogenized for 3 min in a Retsch Knife mill Gm300 (Retsch GmbH, Haan, Germany) at 2000 rotations per minute (rpm), filtered through a sieve (mesh size ~1 mm^2^), centrifuged at 500 rpm for 60 s at 4 °C and the liquid supernatant fraction was collected and frozen at −80 °C as the inoculum.

### Pig faecal extract

Two hundred grams of faeces from each pig were pooled, mixed with 800 mL of sterile 0.9 g/L NaCl solution. The mixture was homogenized for 3 minutes in a Retsch Knife mill Gm300 (Retsch GmbH, Haan, Germany), at 2000 rpm, filtered through a sieve (mesh size ~1 mm^2^) and the liquid fraction was collected. The extract was then centrifuged (3500 rpm, 20 min, 4 °C), the supernatant collected and autoclaved (121 °C, 20 min). The autoclaved extract was centrifuged again (3500 rpm, 20 min, 4 °C) and the supernatant collected and used as a nutrient supplement.

### Strains

The focal species, *E. coli* MG1655, was chromosomally tagged with a Tn*7* gene cassette encoding constitutive red fluorescence, expressed by the *mCherry* gene [20] to ensure that *E. coli* can be detected and distinguished from other community members after competition based on red fluorescence. The Kn resistant, red fluorescent variant containing resistance gene *aph(3*′)-IIb encoding an aminoglycoside 3′-phosphotransferase was created previously [21, 22].

To create the Gm resistant mutant the strain was further tagged through electroporation with the pBAM delivery plasmid containing the mini-Tn*5* delivery system [23, 24] for Gm resistance gene *aacC1* encoding a Gm 3′-*N*-acetyltransferase [25]. Successful clones were screened for Gm resistance (30 μg/mL) and for the chosen clone a single strain growth curve in lysogeny broth (LB) was measured to ensure that the cost of the resistance gene was lower than 10% compared with the susceptible strain to ensure competitive ability.

### Competition experiments

Competition experiments as well as initial growth of focal species strains were performed in 25 mL serum flasks with butyl rubber stoppers. As growth medium 10 mL of sterile LB medium supplemented with 0.1% pig faecal extract, 50 mg/L Cysteine-HCl as an oxygen scrubber and 1 mg/L resazurin as a redox indicator to ensure anaerobic conditions [26], was added to each reactor, heated in a water bath to 80 °C and bubbled with oxygen-free N_2_ gas until the oxygen indicator resazurin turned colourless. After cooling down to 37 °C the appropriate concentration of antibiotic was added from a 1000× anaerobic stock solution.

Two isogenic pairs of the focal species, the susceptible, red fluorescent *E. coli* strain with either its Gm or Kn resistant counterpart, were competed across a gradient of six antibiotics concentrations (Gm [μg/mL]: 0, 0.01, 0.1, 1, 10, 100; Kn [μg/mL]: 0, 0.02, 0.2, 2, 20, 200). Strains as well as the community (100 μL of frozen stock) were grown separately under anaerobic conditions in triplicate reactors, replicates were combined, harvested through centrifugation, washed twice in 0.9% anaerobic NaCl solution and finally resuspended in 0.9% NaCl solution, adjusted to OD_600_ 0.1 (~10^7^ bacteria/mL) and subsequently used in competition experiments. While the community was grown as an inoculum from the same frozen, homogenized stock, both subsampling and cultivation bias, inherent when growing an environmental community under laboratory conditions led to differences in original composition of the model community (Figs. S1, S2). When growing the community in isolation in the absence of antibiotics carrying capacity was reached after 18 h based on OD_600_ readings in a spectrophotometer.

Isogenic strains were mixed at 1:1 ratio (community absent treatment), and that mix further added at 10% ratio to 90% of the faecal community (community present treatment). Approximately 10^6^ bacteria of either mix were transferred to six replicate reactors of each of the antibiotic concentrations and grown at 37 °C with 120 rpm shaking for 24 h, which allowed growth up to carrying capacity. As a consequence of normalizing the total inoculum size the resulting inoculum size of the focal species in absence (~10^6^ bacteria) and presence (~10^5^ bacteria = 10% of total inoculum) of the community differed. A volume of 100 μL of each reactor was then transferred to a fresh bioreactor, grown for 24 h, transferred again for a final 24 h growth cycle and finally harvested for subsequent analysis.

### Fitness assay

From each reactor after 3 days (T_3_), as well as the inocula (T_0_), a dilution series in sterile 0.9% NaCl solution was prepared and plated on LB and LB + AB (30 μg/mL Gm or 75 μg/mL Kn). For appropriate dilutions total and resistant red fluorescent *E. coli* colonies were counted under the fluorescence microscope. Plating of the susceptible strain on LB + AB plates further did not lead to any growth of spontaneous mutants. The relative fitness (*ρ*) of the resistant (*r*) compared with the susceptible strain (*s*) strain was subsequently calculated based on their individual growth rate (*γ*) throughout the competition experiment:

$$\rho 	= \frac{{\gamma _r}}{{\gamma _s}} = \frac{{{\mathrm{log}}\left( {10^6 \times n_r^{T_3}/n_r^{T_0}} \right)}}{{{\mathrm{log}}\left( {10^6 \times n_s^{T_3}/n_s^{T_0}} \right)}} \\ 	= \frac{{{\mathrm{log}}\left( {10^6 \times n_r^{T_3}/n_r^{T_0}} \right)}}{{{\mathrm{log}}\left( {10^6 \times \left( {n_{total}^{T_3} - n_r^{T_3}} \right)/\left( {n_{total}^{T_0} - n_r^{T_0}} \right)} \right)}}$$

Statistical significance testing (*n* = 6) was performed using a one-tailed *t*-test against neutral selection (*ρ* = 1) and ANOVA corrected for multiple testing to compare the relative fitness of different samples.

### MIC assay

To assess the MIC of the susceptible and the resistant focal strain individually in the presence and absence of the microbial community reactors were inoculated with 10^5^ of the focal bacteria and 10^6^ bacteria from the community for the community present treatment. Triplicate reactors were grown overnight across a gradient of antibiotics. Concentrations were increased by 1 μg/mL (Gm susceptible), 2 μg/mL (Km susceptible), 25 μg/mL (Gm resistant) and 50 μg/mL (Km resistant), respectively. Reactors were then harvested and plated out on LB agar. Positive growth was scored as more than fourfold bacterial colonies growing on the plates compared with plating of the inoculum. The MIC was defined as the first concentration at which no positive growth was observed.

### DNA extraction and sequencing

Bacteria from each reactor, as well as inoculum and original pig faecal community were harvested through centrifugation of 2 mL of liquid, followed by DNA extraction using the Qiagen PowerSoil kit as per the manufacturer’s instructions. The quality and quantity of the extractions was confirmed by 1% agarose gel electrophoresis and dsDNA BR (Qubit), respectively.

16S rRNA gene libraries were constructed using multiplex primers designed to amplify the V4 region [27]. Amplicons were generated using a high-fidelity polymerase (Kapa 2G Robust), purified with the Agencourt AMPure XP PCR purification system and quantified using a fluorometer (Qubit, Life Technologies, Carlsbad, CA, USA). The purified amplicons were pooled in equimolar concentrations based on Qubit quantification. The resulting amplicon library pool was diluted to 2 nM with sodium hydroxide and 5 mL were transferred into 995 mL HT1 (Illumina) to give a final concentration of 10 pM. Six hundred millilitres of the diluted library pool was spiked with 10% PhiXControl v3 and placed on ice before loading into Illumina MiSeq cartridge following the manufacturer’s instructions. The sequencing chemistry utilized was MiSeq Reagent Kit v2 (500 cycles) with run metrics of 250 cycles for each paired end read using MiSeq Control Software 2.2.0 and RTA 1.17.28.

Metagenomic libraries were created using the KAPA high throughout Library Prep Kit (Part no: KK8234) optimized for 1 μg of input DNA with a size selection and performed with Beckman Coulter XP beads (Part no: A63880). Samples were sheared with a Covaris S2 sonicator (available from Covaris and Life Technologies) to a size of 350 bp. The ends of the samples were repaired, the 3′–5′ exonuclease activity removed the 3′ overhangs and the polymerase activity filled in the 5′ overhangs creating blunt ends. A single ‘A’ nucleotide was added to the 3′ ends of the blunt fragments to prevent them from ligating to one another during the adapter ligation reaction. A corresponding single ‘T’ nucleotide on the 3′ end of the adapter provided a complementary overhang for ligating the adapter to the fragment ensuring a low rate of chimera formation. Indexing adapters were ligated to the ends of the DNA fragments for hybridisation on a flow cell. The ligated product underwent size selection using the XP beads detailed above, thus removing the majority of un-ligated or hybridized adapters. Prior to hybridisation the samples underwent six cycles of PCR to selectively enrich those DNA fragments with adapter molecules on both ends and to amplify the amount of DNA in the library. The PCR was performed with a PCR primer cocktail that anneals to the ends of the adapter. The insert size of the libraries was verified by running an aliquot of the DNA library on a PerkinElmer GX using the High Sensitivity DNA chip (Part no: 5067–4626) and the concentration was determined by using a High Sensitivity Qubit assay. All raw sequencing data have been submitted to ENA under study accession number PRJEB29924.

### 16S rRNA gene analysis

Sequence analysis was carried out using mothur v.1.32.1 [28] and the MiSeq SOP [27] as accessed on 07.08.2017 on http://www.mothur.org/wiki/MiSeq_SOP. Sequences were classified based on the RDP classifier [29]. Diversity was assessed based on observed OTUs at 97% sequence similarity. All sequences of the focal species *E. coli* were removed based on ≥99% sequence similarity. No sequences with this degree of similarity to the focal species were detected in the original faecal community. NMDS plots for the community were created based on the Bray–Curtis dissimilarity metric [30].

Further sample similarity was tested using analysis of molecular variance (AMOVA) a nonparametric analogue of traditional ANOVA testing. AMOVA is commonly used in population genetics to test the hypothesis that genetic diversity between two or more populations is not significantly different from a community created from stochastically pooling these populations [31, 32].

### Metagenomic analysis

Metagenomic samples, as well as a reference genome for the focal species *E. coli* MG1655, were analysed using the ARG-OAP pipeline for antibiotic resistance genes detection from metagenomic data using an integrated structured antibiotic resistance gene database [33]. This resulted in the abundance of different resistance gene classes and subtypes within these groups normalized by 16S rRNA gene copy number. Antibiotics resistance genes detected in the *E. coli* reference genome were subtracted from the total number of hits per 16S rRNA gene copy based on the abundance of *E. coli* 16S rRNA gene/total 16S rRNA gene. Further, all antibiotics resistance gene numbers were normalized to the amount of pig faecal community 16S rRNA gene per total 16S rRNA gene copy.

### Mathematical model

In order to illustrate possible mechanisms underlying the data for bacterial fitness in the presence/absence of the community for varying concentrations of Gm and Kn, we described our experimental setup mathematically. For this we first developed a discrete-time mathematical model for the growth of the susceptible and drug-resistant bacteria, *s* and *r*, respectively, in the presence or absence of the community, *c*.

#### Bacterial growth

The discrete-time model describing the growth of the bacteria *i*, *i* = *s, r, c*, is governed by the following iterative model

$$n_i^{t + 1} = n_i^t\left( {1 + \phi _i\left( {1 - g_i} \right)\left( {1 - f_i} \right)} \right),$$

where $n_i^{t + 1}$ is the size of the population of strain *i* at time *t* + 1, and *ϕ*_*i*_ is the maximum growth rate in the absence of competition and drug pressure. The reduction in growth due to density-dependent regulation/resource limitation, given as

$$g_i = \frac{{\mathop {\sum }\nolimits_j e_{ij}n_j}}{{k_d}},$$

with *k*_*d*_ as the carrying capacity and *e*_*ij*_ being the competition coefficient, describing how much the presence of an allospecific strain *j* impacts the competitive fitness of strain *i*. The reduction in bacterial growth due to drug pressure, *f*_*i*_, is governed by a generalised logistic function

$$f_i = {\mathrm{min}}\left( {f_{{\mathrm{max}}},\frac{1}{{1 + e^{\alpha _i - \beta _i\ln c}}}} \right),$$

where *c* is the drug concentration (in μg/mL), α_*i*_ and β_*i*_ are the parameters describing the dose-response relationship for strain *i*, and *f*_max_ = 0.9 is the maximum growth inhibition.

#### Model simulation and relative fitness calculation

Starting from an initially small number of bacteria in fresh medium, we ran the model for 30 generations, at which point the bacterial population had reached carrying capacity, and diluted the population accordingly. The bacteria were again allowed to grow for 30 generations before being diluted and grown for a final 30 generations. At this point we calculated the relative fitness of the resistant strain as

$$\rho = \frac{{\gamma _r}}{{\gamma _s}} = \frac{{{\mathrm{log}}\left( {10^6 \times n_r^{90}/n_r^0} \right)}}{{{\mathrm{log}}\left( {10^6 \times n_s^{90}/n_s^0} \right)}}.$$

#### Community-dependent change in drug resistance/susceptibility

The Kn data seem to suggest that the benefit of the drug resistant bacteria is reduced in the presence of the community at medium to high drug concentrations pointing towards a decrease in the susceptibility of the susceptible strain in a community context. We captured this scenario by making the dose-response parameters *α*_*s,r*_ and *β*_*s,r*_ explicitly dependent on the density of the community by increasing the resistance of susceptible strain, *s*, i.e.

$$\alpha _s\left( t \right) = \alpha _{s,0}\left( {1 + \frac{{1.3\,n_c^t}}{{n_c^t + 10^3}}} \right),$$

$$\beta _s\left( t \right) = \beta _{s,0}\left( {1 + \frac{{0.35\,n_c^t}}{{n_c^t + 10^3}}} \right),$$

where α_*i,0*_ and β_*i,0*_ are the time-independent dose-response parameters. The effect of density dependence is further illustrated (Fig. S3).

### Parameter estimations

For each drug (Gm and Kn) we obtained a set of parameter values that resulted in a good overall fit between the model simulations and the data, where the data comprised the observed relative fitness for both sets of experiments (i.e. bacteria grown in the presence and absence of the community) for six different drug concentrations. To allow for logarithmic regression the non-antibiotic control was assumed as one order of magnitude lower than the lowest concentration used in the experiment. The parameter values were determined by minimising the root-mean-square error using an optimisation algorithm akin to simulated annealing [34]. The aim here was not to perform rigorous parameter estimation but rather to find a set of parameters that, given specific model constraints and assumptions, resulted in model behaviours that qualitatively agreed with both the observed dynamics over the repeated growth cycles and the empirically determined fitness values. In fact, our method failed to find a unique set of values that consistently gave the best fitting model, which suggests that the available data was insufficient to determine the global maximum. However, the qualitative relationships between individual parameters and between the parameters comparing the two antimicrobials were fairly consistent between model runs. Tables 1, 2 list the sets of parameters estimated for the two different antibiotics. These parameters allow changes in community and focal species densities to be estimated throughout the individual competition experiments based on start- and end-point measurements (Fig. S4).

**Table 1:** Model parameter values for gentamicin selection curves

Gentamicin				
Parameter	All	Susceptible	Resistant	Community
*φ*		1.4	1.3	1.3
*e*_*ij*_		1	2.3	3
*α*_i,0_		1.3	2.9	1.6
*β*_i,0_		0.7	0.8	0.6
*k*_*d*_	10^5^	–	–	–
*f*_max_	0.9	–	–	–

**Table 2:** Model parameter values for kanamycin selection curve

Kanamycin				
Parameter	All	Susceptible	Resistant	Community
*φ*	–	1.9	1.8	1.3
*e*_*ij*_	–	1.7	1.3	1.6
*α*_i,0_	–	1.0	1.4	1.6
*β*_i,0_	–	0.6	0.4	0.5
*k*_d_	10^5^	–	–	–
*f*_max_	0.9	–	–	–

## Results

### Community context affects selection for gentamicin resistance

**Fig. 1:**
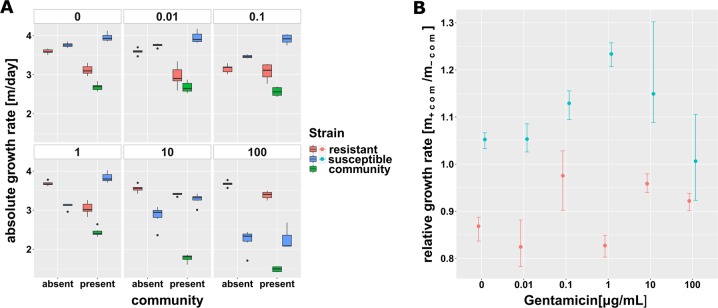
Malthusian growth parameter per day of the focal species’ isogenic strains for gentamicin. Values are displayed across the antibiotic gradient and in absence and presence of the gut microbial community. **a** Average (±SD, *n* = 6) logarithmic absolute growth per day for the resistant strain, the susceptible strain and the community. A different inoculum size of the focal species in absence (~10^6^ bacteria) and presence (~10^5^ bacteria = 10% of total inoculum) of the community was used. **b** Ratio of absolute Malthusian growth parameters (with 95% confidence intervals based on 1000-fold bootstrap analysis) in presence and absence of the microbial community across the gradient of antibiotic concentrations

Isogenic strains of the focal species *E. coli*, with and without Gm resistance, were competed in the presence and absence of a pig faecal community across a 5 orders of magnitude gradient of Gm concentrations. Independent of antibiotic concentration the focal species increased in abundance during the 3 day competition experiment from ~10% at inoculation to above 90% relative abundance based on 16S rRNA gene sequencing (Fig. S5A and B). Both resistant and sensitive strains, as well as the community, showed positive growth across the whole range of concentrations and both treatments with focal species’ cell counts increasing by 2.25–3.96 orders of magnitude per day (Fig. 1a).

**Fig. 2:**
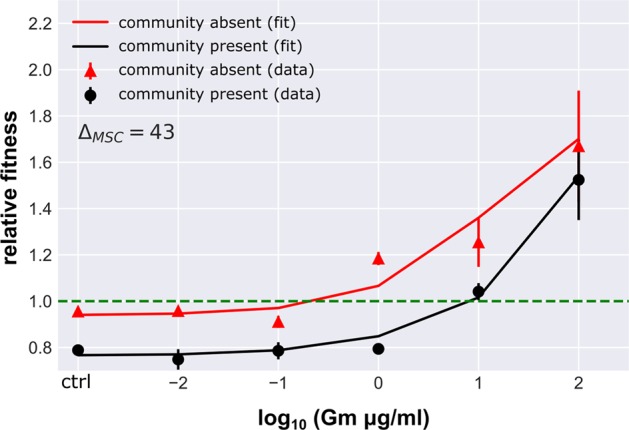
Relative fitness of the gentamicin resistant strain. Values (mean ± SD, *n* = 6) in presence (black) and absence (red) of the community. Solid lines represent the best fit fitness curve through the mathematical model based on parameter estimates presented in Table 1. The dashed line indicates neutral selection at a relative fitness of *ρ*_r_ = 1, where the intercept with the fitness curve indicates the minimal selective concentration

There was a small competitive fitness cost (*t*-test against 1, *p* = 0.0005) of Gm resistance in the absence of the community (*ρ*_r_ = 0.955 ± 0.014, mean ± SD), and this cost appeared to be greatly increased when the community was present (Fig. 2) (*ρ*_r _= 0.788 ± 0.016) (ANOVA corrected for multiple testing, *p* < 0.01, *F* = 360.36). At all concentrations between 0 and 10 μg/mL the susceptible strains relative growth benefited more from presence of the community when compared with the resistant one (ANOVA corrected for multiple testing, *p* < 0.05), until at 100 μg/mL Gm, the community had no significant effect on relative growth (ANOVA corrected for multiple testing, *p* = 0.259, *F* = 1.42) (Fig. 1b).

#### Community composition is altered across the gentamicin gradient

It is possible that changes in community composition across the antibiotic gradient may have contributed to the observed changes in selection for resistance caused by the community, notably between 10 and 100 μg/mL. The composition of the microbial community changed significantly from the collected faecal sample, to inoculum and further during the duration of the experiment (AMOVA, *p* < 0.001, Fig. S1A). Above 1 μg/mL Gm the previously dominant *Proteobacteria* were outcompeted by *Firmicutes* (Fig. S1B) leading to a significant (AMOVA, *p* < 0.01) separation of communities below and above this threshold concentration in the NMDS plot (Fig. S1A). However, there was no significant change in composition between 10 and 100 μg/mL, suggesting that compositional changes did not play a major role in community-imposed selection.

#### Community context imposes a cost of resistance

To test the hypotheses derived from the numerical data we used numerical simulations of our experimental set up to determine the likely mechanisms underpinning the observed population dynamics in a common logarithmic growth model. We determined models based on the key empirical findings in the absence of the community (specifically, that there is a cost of resistance in the absence of antibiotics, and that antibiotics inhibit the growth of the sensitive strain in a dose dependent manner), and then determined the most parsimonious way, in which the community could have altered the relative fitness of the resistant and susceptible strains (Table 1). We found a good fit to the data simply by assuming that the community imposed a greater competitive effect, constant across the antibiotic gradient, on the resistant rather than the sensitive strain (*e*_*rj*_ >> *e*_*cj* _> *e*_*sj*_; where *e*_*ij*_ is the competition coefficient imposed on the focal population (resistant *r*, susceptible *s* and community *c*) by the community). Further, higher concentrations of Gm result in a drastic drop in the community’s growth rate, and hence a reduction of the elevated cost of resistance imposed by the community at these higher levels of antibiotic due to reduced competition. This in combination with very little growth of the susceptible strain explains why the relative fitness between the community present and community absent treatments converged at high antibiotic concentrations (100 μg/mL) with relative fitness primarily determined by the growth of the resistant strain.

The numerical simulation allowed us to estimate the change in MSC from absence to presence of the community by deterministically evaluating the concentration at the intercept with neutral selection at a relative fitness of *ρ*_r_ = 1. We estimated a 43-fold increase in MSC in the presence of the community (Fig. 2). The MIC for the susceptible strain remained stable at 8–9 μg/mL in both presence and absence of the community (Table 3). Consequently, this 43-fold increase in MSC shifts selection from concentrations far below the MIC in absence to concentrations around the MIC in presence of the community.

**Table 3:** MIC of the focal species, in isolation in presence and absence of the community. The range of highest growth concentration to minimal tested inhibitory concentration is shown

Antibiotic	Strain	Community	MIC [μg/mL]
Gentamicin	Susceptible	Absent	8–9
		Present	8–9
	Resistant	Absent	100–125
		Present	100–125
Kanamycin	Susceptible	Absent	16–18
		Present	18–20
	Resistant	Absent	>800
		Present	>800

### Community context affects selection for kanamycin resistance

**Fig. 3:**
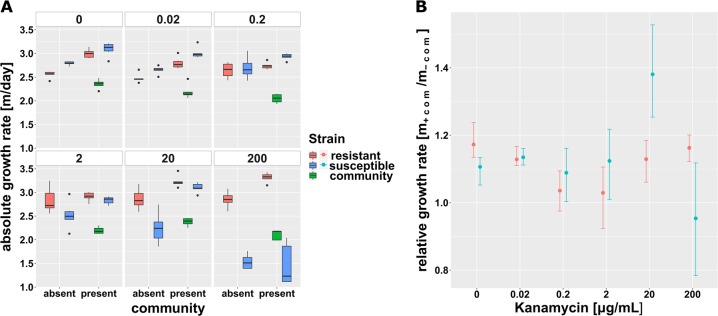
Malthusian growth parameter per day of the focal species’ isogenic strains for kanamycin. Values are displayed across the antibiotic gradient and in absence and presence of the gut microbial community. **a** Average (±SD, *n* = 6) logarithmic absolute growth per day for the resistant (red), the susceptible strain and the community. A different inoculum size of the focal species in absence (~10^6^ bacteria) and presence (~10^5^ bacteria = 10% of total inoculum) of the community was used. **b** Ratio of absolute Malthusian growth parameters (with 95% confidence intervals based on 1000-fold bootstrap analysis) in the presence and absence of the microbial community across the gradient of antibiotic concentrations

As with Gm, the focal species increased in abundance during the 3 day competition experiment from ~10% at inoculation to above 90% relative abundance (Fig. S5C and D). Again, both strains, as well as the community, increased in abundance across both treatments and all concentrations of the 5 orders of magnitude antibiotic gradient with focal species’ cell numbers increasing by 1.45–3.09 orders of magnitude per day (Fig. 3a). In the absence of this community, Kn resistance also imposed a slight metabolic fitness cost on the resistant strain (*ρ*_r _= 0.915 ± 0.036) (Fig. 3b).

**Fig. 4:**
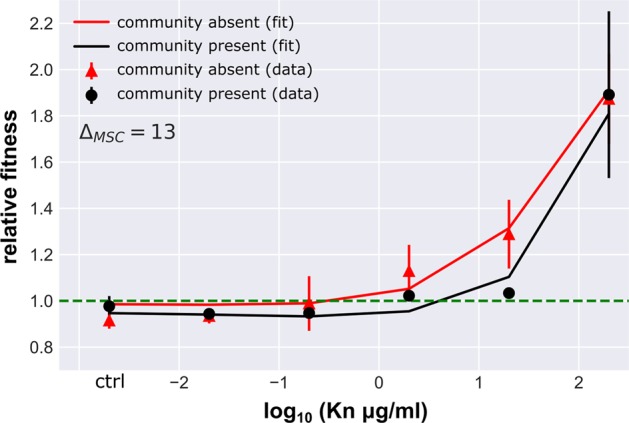
Relative fitness of the kanamycin resistant strain. Values (mean ± SD, *n* = 6) in the presence (black) and absence (red) of the community. Solid lines represent the best fit fitness curve through the mathematical model based on parameter estimates presented in Table 2. The dashed line indicates neutral selection at a relative fitness of *ρ*_r_ = 1, where the intercept with the fitness curve indicates the minimal selective concentration

However, unlike Gm, the community did not increase the general cost of resistance. Indeed, the community had no significant effect on the relative fitness of the resistant strain except at a concentration of 20 μg/mL (ANOVA corrected for multiple testing, *p* = 0.002, *F* = 15.58) (Fig. 4). There was a clear fitness advantage for the resistant strain in the absence of the community at this concentration (*ρ*_r _= 1.288 ± 0.149; *t*-test against 1, *p* = 0.0052), while in the presence of the community, this difference in relative fitness, though still significant (*t*-test against 1, *p* = 0.0088), was considerably lower (*ρ*_r _= 1.034 ± 0.020). At 200 μg/mL Kn, close to the susceptible strains MIC, the resistant strain had an equally high relative fitness regardless of the presence of the community (ANOVA corrected for multiple testing, *p* = 0.079, *F* = 3.84).

*Community and antibiotic resistance composition remain stable across the kanamycin gradient*

**Fig. 5:**
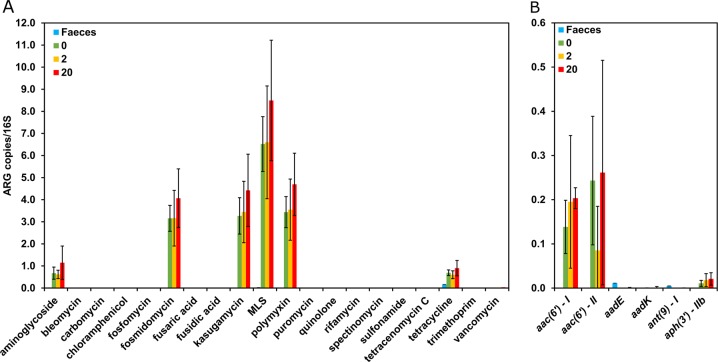
Detected resistance genes. Type (**a**) and aminoglycoside subtype (**b**) relative abundance (resistance gene number normalized with 16S rRNA gene copy number), in original faecal community and in final reactor community at three kanamycin concentrations (mean ± SD; *n*_faeces_ = 2, *n*_Kn0_ = 6, *n*_Kn2_ = 6 *n*_Kn20_ = 5). Only genes detected with the ARGs-OAP pipeline are shown. MLS = Macrolides, Lincosamides, Streptogramines

As with the Gm experiment, a significant shift in community composition from collected faecal sample, to inoculum and further during the duration of the Kn experiment (AMOVA, *p* < 0.001, Fig. S2A) was observed. However, across the whole gradient of antibiotics, *Firmicutes* (Fig. S2B) remained the dominant phylum with no significant changes in community composition as a result. As such, compositional changes again cannot explain the impact of the community on focal strain fitness under selection at 20 μg/mL only. We additionally carried out metagenomic analysis for the 0, 2 and 20 μg/mL Kn treatments to determine whether relative abundance of resistance genes had changed within the community, despite the fact that there were no changes in community composition. Resistance to aminoglycoside (ANOVA, *p* = 0.04) and other classes of antibiotics (fosmidomycin, kasugamycin, macrolides, polymyxin and tetracycline (ANOVA, all *p* < 0.01)) significantly increased in the community of all reactors compared with the original faecal community independent of antibiotic concentrations (Fig. 5a). However, there was no significant difference between Kn concentration and the abundance (ANOVA, *p* = 0.15) of aminoglycoside resistance in general (Fig. 5a) or any specific aminoglycoside resistance subtypes (Fig. 5b) suggesting that relative fitness of the focal species was not influenced by the community resistome. Resistance genes detected by metagenomic analysis were however expressed, since resistant colonies from community members were detected on selective plates after the competition experiment. Unsurprisingly, since no antibiotic concentration-dependent selection for aminoglycoside resistance was observed within the community, no significant co-selection for resistance to any other classes of antibiotics was observed either.

*Presence of the community can enhance growth of susceptible E. coli population at intermediate antibiotic concentrations*

Numerical simulations showed that, unlike for Gm resistance, a community-imposed increase in the cost of Kn resistance was unable to explain why the benefit to the drug resistant focal *E. coli* strain was reduced in the presence of the community at intermediate drug concentrations (*e*_*rj*_ = *e*_*s*_). This suggested different interactions between *E. coli* and the rest of the community, and we speculated that the community might have provided a protective effect against Kn for the susceptible *E. coli*. Growth data demonstrated this to be the case: in the community treatment the growth rate of both the susceptible and resistant *E. coli* was altered in a consistent fashion across the lower concentrations. The only exceptions was observed at 20 μg/mL, where the growth rate of the susceptible, but not the resistant strain, was significantly increased by the presence of the community (ANOVA corrected for multiple testing, *p* = 0.002, *F* = 15.58) (Fig. 3b). Further, a slight increase in the MIC of the susceptible strain in the presence of the community from 16–18 to 18–20 μg/mL (Table  3) supports the notion of a protective effect. We investigated if a protective effect of the community was sufficient to explain the observed data by fitting numerical simulations where the dose-response parameters *α*_*s,r*_ and *β*_*s,r*_ were explicitly dependent on the time-dependent density of the community (as listed in Table 2). The resulting model provided a good fit to the experimental data, suggesting that community protection was driving the observed population dynamics with a 12-fold increase in MSC. Despite this MSC shift, other than for Gm resistance for Kn selection was still observed at concentrations 5–10-fold below the MIC of the susceptible strain.

## Discussion

In this study we investigated how being embedded within a semi-natural community (a pig gut derived community in an anaerobic digester) affects selection for AMR within a focal species (*E. coli*). For two antibiotics (Gm and Kn), we find the presence of the community selects against resistance, resulting in 1–2 orders of magnitude higher minimal selective concentrations for antibiotic resistance and thus achieving selection either closer towards or for Gm even at around the susceptible strains MIC. This suggests that recent in vitro single strain based estimates of MSCs [6, 7, 11] are likely much lower than would be observed in vivo and might explain why in certain ecosystems no selection for antibiotic resistance was observed in focal strains [35].

The primary mechanisms responsible for this community-imposed reduction in selection for resistance differed for the two tested drugs, yet are likely fairly general based on their ecological origin. For Gm, the community increased the fitness costs reflected by reduced growth rates that are associated with resistance in the absence of antibiotics. These elevated costs were retained at similar levels across the antibiotic gradient, up until doses were so high that only the resistant strain grew (similar behaviour above a certain threshold concentration has previously been described for single strain systems [36, 37] and our results show that this holds true in a community context). Resource limitation—directly manipulated or through competition—has been found to increase costs against a range of stressors in a range of organism, from resistance of plasmodium to antimalarial drugs [38] to phage resistance in bacteria [18]. This is presumably because resource limitation has a more pronounced effect on resistant genotypes [39].

For Kn, community-imposed selection against resistance was only apparent at intermediate antibiotic concentrations. The absolute growth rate of the susceptible strain was significantly increased at intermediate concentrations in presence of the community. Our model fitting suggests this is because of a protective effect of the community, further supported by a slight increase in the MIC of the susceptible strain in presence of the community. The protective effect might have only been observed at intermediate concentrations since low concentrations were insufficient to detectably lower the relative fitness of the susceptible strain, while at high concentrations the protective effect was too small to be detectable. Such protective effects have been reported extensively within species [13, 14], as well as more recently within more complex communities [12], either because of extra- or intracellular modification of antibiotics. Other common mechanisms known to increase a strains resistance to antibiotics in communities involve flocculation [40] or biofilm formation [41, 42], but might here only play a minor role due to the shaking conditions.

A main difference between the Kn and Gm competition experiments was observed that could have implications for the likely mechanisms underpinning community-mediated changes in selection for resistance. In the absence of antibiotic selection, a higher community diversity involving a larger proportion of *Proteobacteria* was detected in the competing community during the Gm experiment compared with the Kn experiment. Since the focal species *E. coli* also belongs to the *Proteobacteria*, the difference in community structure and especially proteobacterial abundance could have increased the level of niche overlap between focal strain and community and thus competition [43], which may explain why Gm resistance had a significantly increased cost while Kn resistance did not.

The two general mechanisms discussed above all underlie selection for standing variation in pre-existing resistance genes, rather than selection on de novo variation arising through spontaneous mutations or horizontal gene transfer from other species. For de novo chromosomal mutations, the community is likely to further limit the spread of resistance, because the reduced population sizes of the focal strains in the presence of the community increase the chance that more costly mutations will be fixed [44]. In contrast, being embed in a community might enhance the spread of resistance. First, there will be a greater source of resistance genes available to the focal species. Second, selection against resistance acquired through horizontal gene transfer at low antibiotic concentrations might follow different dynamics. While chromosomal resistance might be outcompeted and subsequently lost, resistance genes embedded on conjugative plasmids can persist or even increase in abundance, as a consequence of their sometimes extremely broad host ranges and high transfer frequencies [22, 45–48]. In controlled single strain experiments plasmid born resistance proved more costly than chromosomal resistance [7]. However, in more complex scenarios selection for mobile genetic element borne resistance usually depends not only on the single acquired resistance gene, but a combination of other linked traits encoded by the MGE as part of the communal gene pool [49]. Thus, difficulties in making general predictions on the selection dynamics of horizontally acquired resistance in microbial communities arise that merit future research efforts.

In summary, we show that selection for AMR was influenced by being embed in a ‘natural' microbial community, such that the MSC was increased by more than one order of magnitude for two different antibiotics. Further to reducing relative fitness of resistance, being embedded in a community would also reduce absolute fitness, which has been argued to sometimes be the major driver of spread of resistance [50]. The aim of this study was to identify these general mechanisms underlying decreased selection for AMR in complex community context with a high degree of environmental and ecological realism. More specific individual interactions contributing to these general effects might however depend on the specific community, antibiotic and corresponding resistance genes and should be investigated in the future.

To determine MSCs that are relevant in environmental settings it is thus crucial to test for selection in a complex community context, rather than in single strain systems. Understanding under which concentrations selection for and thus long-term fixation of newly acquired resistance mechanisms is occurring is crucial for future mitigation of the spread of resistance genes as well as their potentially pathogenic hosts [51, 52]. Our results further stress the need to preferentially use narrow spectrum antibiotics in clinical therapy to maintain a healthy microbiome within the patient that can more easily recover after antibiotic administration [53], thus decreasing the likelihood of positive selection for pathogens that might have acquired resistance when embedded in a community.

## Supplementary information

Supplementary Text

Figure S1

Figure S2

Figure S3

Figure S4

Figure S5
