# Supplementary material for: Selection for antimicrobial resistance is reduced when embedded in a natural microbial community
Source: ISME J. 2019 Aug 5;13(12):2927–37. doi: 10.1038/s41396-019-0483-z (PMC6864104; doi:10.1038/s41396-019-0483-z)
Supplement: Supplementary file 2 — Figure S1 [file 41396_2019_483_MOESM2_ESM.docx]

## Supplementary Information

## Title

Selection for antibiotic resistance is reduced when embedded in a natural microbial community

## Author list

Uli Klümper^1,2,*^, Mario Recker^3^, Lihong Zhang^2^, Xiaole Yin^4^, Tong Zhang^4^, Angus Buckling^1^, William H. Gaze^2^

^1^ CLES & ESI, University of Exeter, Penryn, Cornwall, United Kingdom

^2^ European Centre for Environment and Human Health, University of Exeter Medical School, ESI, Penryn, Cornwall, United Kingdom

^3^ College of Engineering, Mathematics and Physical Sciences, University of Exeter, Penryn, Cornwall, United Kingdom

^4^ Department of Civil Engineering, University of Hong Kong, Hong Kong, China

^*^corresponding author:

Uli Klümper

CLES & ESI University of Exeter

TR109FE Penryn

United Kingdom

Email: [u.klumper@exeter.ac.uk](mailto:u.klumper@exeter.ac.uk)

Phone: (+44)7497497338

ORCID: 0000-0002-4169-6548

**Figure Legends**

**Figure SI1. Community analysis for gentamicin reactors.**

(A): Non-metric 2-dimensional scaling analysis (NMDS) revealing distinct clustering of original fecal community, inoculum and reactors after 3 day incubation. Ordination based on the Bray-Curtis dissimilarity metric. (B) Bar chart based on phylum distribution.

**Figure SI2. Community analysis for kanamycin reactors.**

(A) Non-metric 2-dimensional scaling analysis (NMDS) revealing distinct clustering of original fecal community, inoculum and reactors after 3 day incubation. Ordination based on the Bray-Curtis dissimilarity metric. (B) Bar chart based on phylum distribution.

**Figure SI3. Contour plots showing the growth reduction of the susceptible strain**,

dependent on the Kanamycin concentration and size of the community, for the two scenarios used in the mathematical model: density independent (a) and dependent (b) resistance

**Figure SI4. Simulated community dynamics.**

Illustrative example of modelled community dynamics (here for Kanamycin 20 μg/mL). Changes in abundance of both focal strains as well as the community through the course of the experiment are displayed in combination with two transfers. Simulation was performed using the parameters gained from the model based on start and end-point densities.

**Figure SI5. Relative abundance of the focal species after 3 days of the competition experiment.**

Values shown are mean ± SD (n=6, resistant: black, susceptible: grey). (A) gentamicin, absolute values including community; (B) gentamicin, ratio of the isogenic pair of the focal species (C) kanamycin, absolute values including community; (D) kanamycin, ratio of the isogenic pair of the focal species.
