# Supplementary figures and images for: Selection for antimicrobial resistance is reduced when embedded in a natural microbial community
Source: ISME J. 2019 Aug 5;13(12):2927–37. doi: 10.1038/s41396-019-0483-z (PMC6864104; doi:10.1038/s41396-019-0483-z)

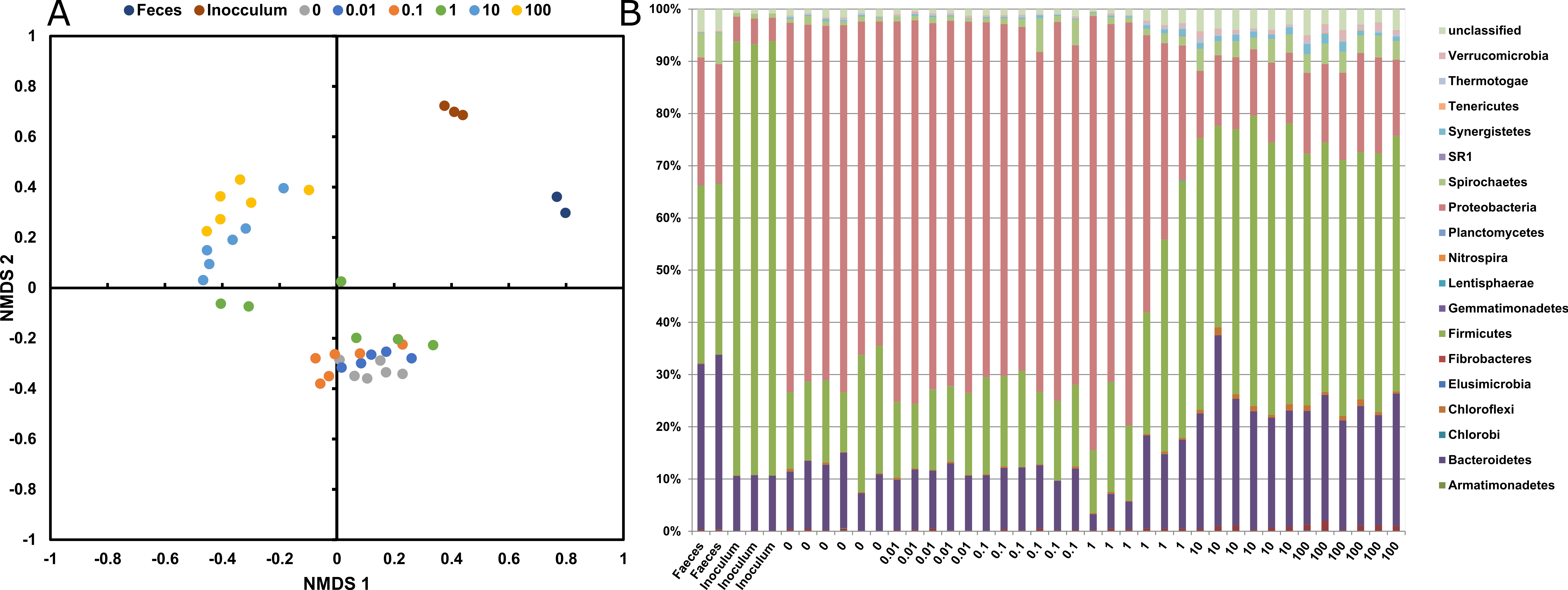

Supplement: Supplementary file 3 — Figure S2 [file 41396_2019_483_MOESM3_ESM.tif]

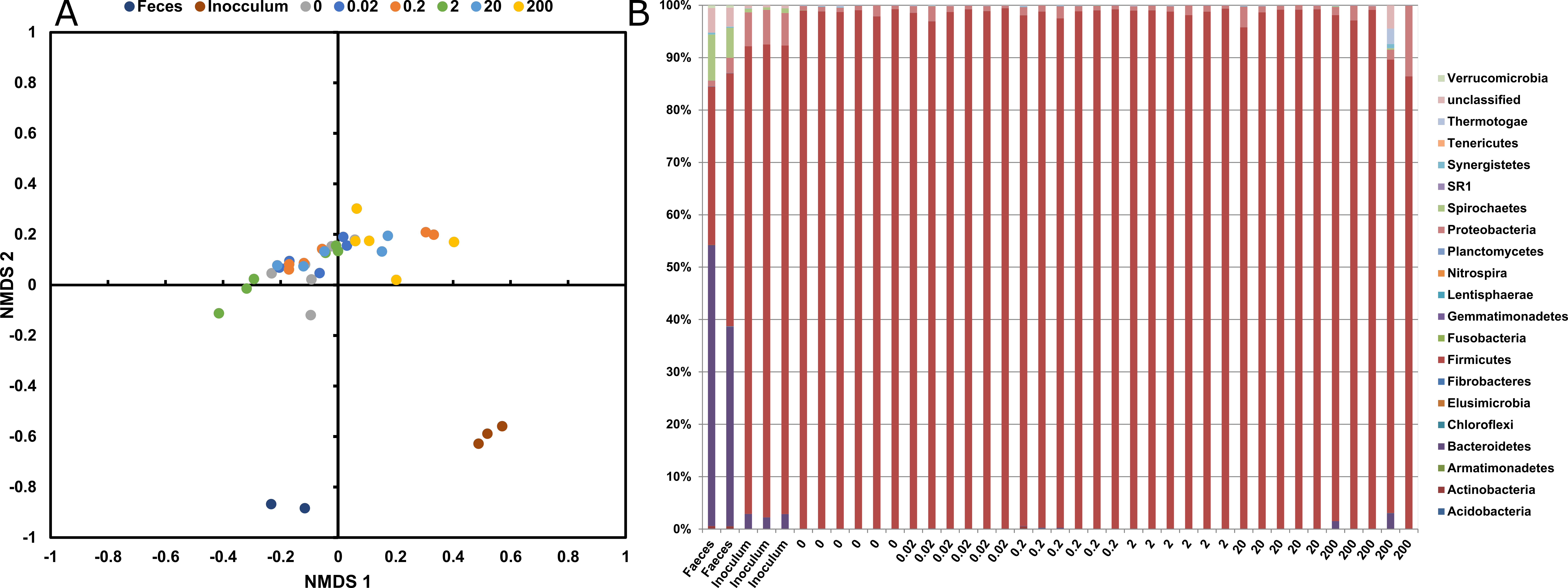

Supplement: Supplementary file 4 — Figure S3 [file 41396_2019_483_MOESM4_ESM.tif]

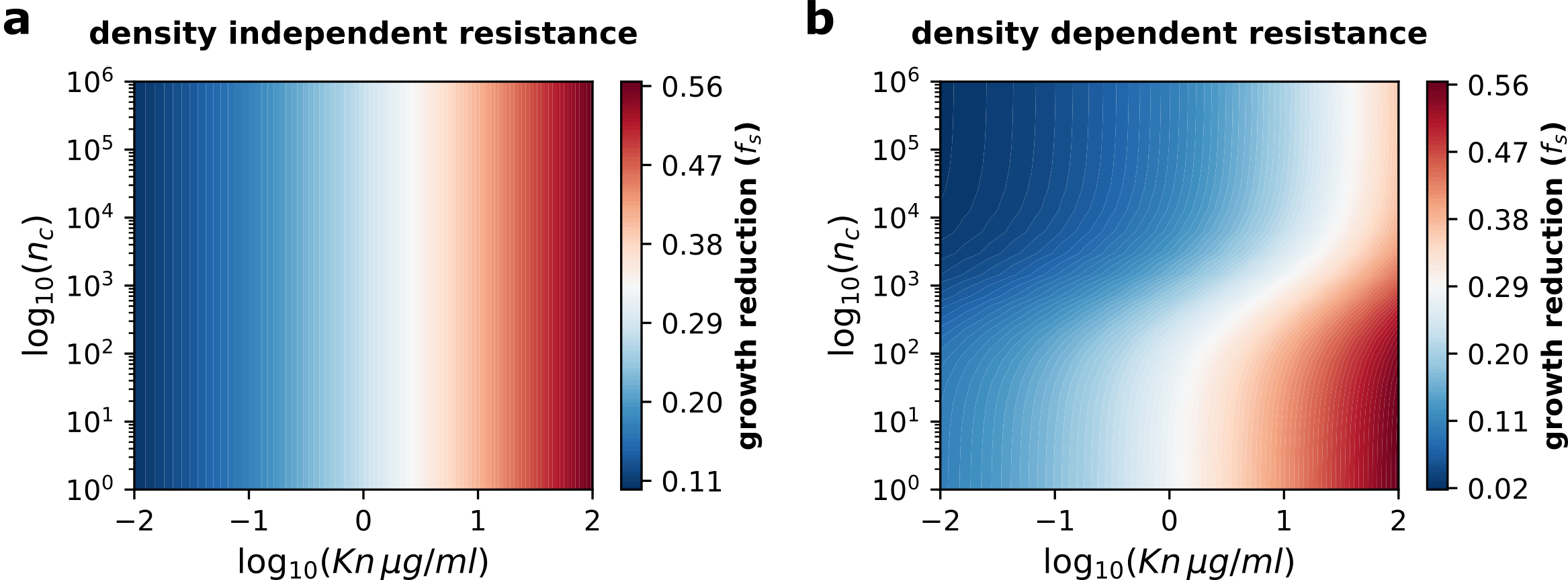

Supplement: Supplementary file 5 — Figure S4 [file 41396_2019_483_MOESM5_ESM.tif]

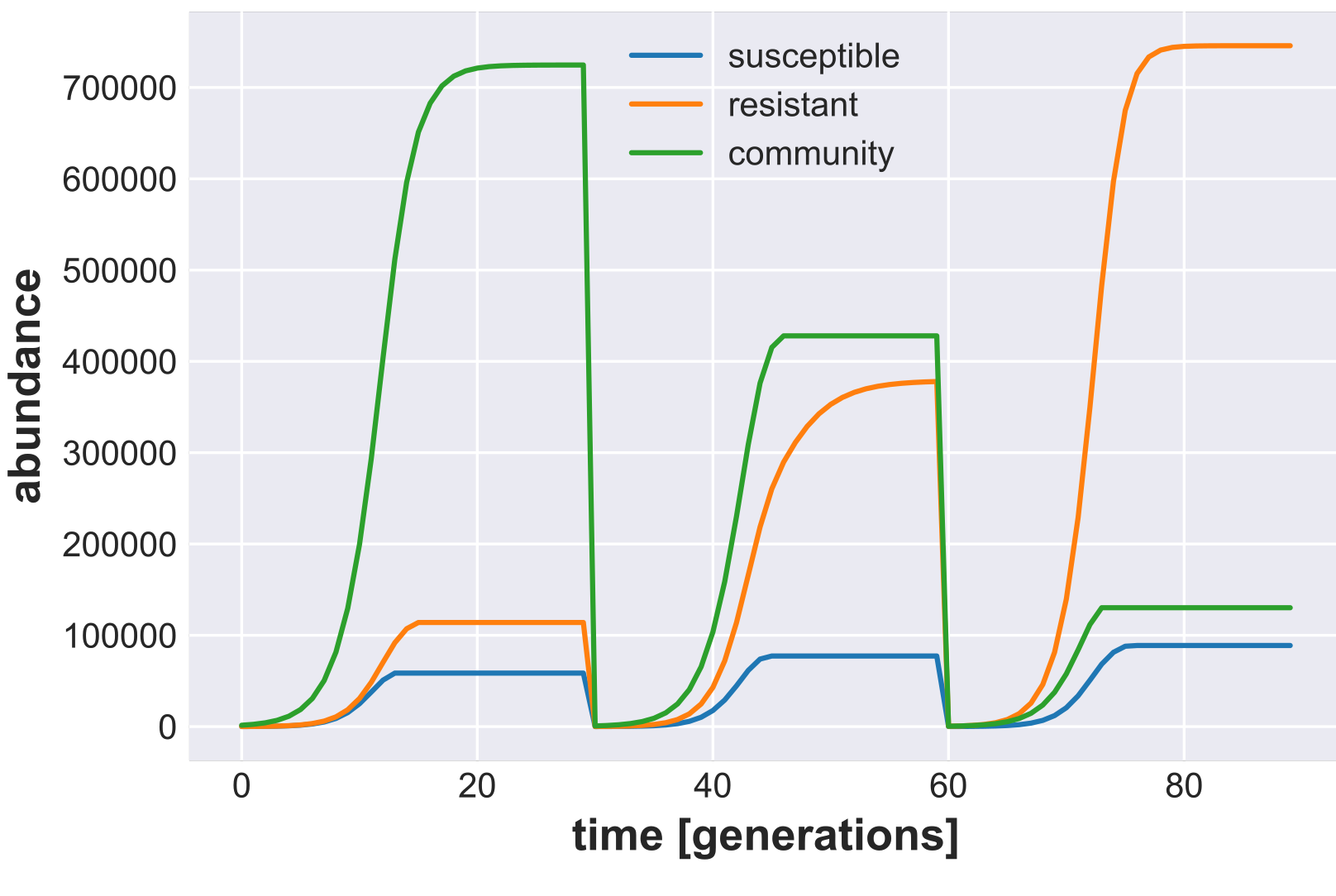

Supplement: Supplementary file 6 — Figure S5 [file 41396_2019_483_MOESM6_ESM.tif]
